# Differential Effects of Intranasal Vasopressin on the Processing of Adult and Infant Cues: An ERP Study

**DOI:** 10.3389/fnhum.2018.00329

**Published:** 2018-08-15

**Authors:** Xiaoyan Wu, Pengfei Xu, Yue-Jia Luo, Chunliang Feng

**Affiliations:** ^1^Center of Brain Disorder and Cognitive Sicences, College of Psychology and Sociology, Shenzhen University, Shenzhen, China; ^2^Shenzhen Key Laboratory of Affective and Social Cognitive Science, Shenzhen University, Shenzhen, China; ^3^Center for Emotion and Brain, Shenzhen Institute of Neuroscience, Shenzhen, China; ^4^College of Information Science and Technology, Beijing Normal University, Beijing, China; ^5^State Key Laboratory of Cognitive Neuroscience and Learning, Beijing Normal University, Beijing, China

**Keywords:** vasopressin, event-related potential (ERP), infant cues, emotion, faces

## Abstract

Arginine vasopressin (AVP) is a powerful regulator of various social behaviors across many species. However, seemingly contradictory effects of AVP have been found in both animal and human studies, e.g., promoting aggression on one hand and facilitating social bonding on the other hand. Therefore, we hypothesize that the role of AVP in social behaviors is context-dependent. To this end, we examined the modulatory effect of AVP on male’s behavioral and neural responses to infant and adult cues. After intranasal and double-blind treatment of AVP or placebo, male participants were asked to rate their subjective approaching willingness to infant and adult faces in specific contexts informed by cue words while EEG recording. Our results showed that AVP treatment increased approaching ratings to neutral and positive other-gender adult faces compared to emotional matched same-gender adult faces, and to negative girl faces compared to negative boy faces. Furthermore, compared to placebo treatment, AVP treatment induced larger N1 amplitudes to neutral cues associated with both adults and infants, whereas AVP treatment only sustained pronounced late positive potential amplitudes to neutral cues of infants but not adults. Those findings implicate differential roles of AVP in the processing of adult- and infant-related cues and thus lend support to the context-dependent account.

## Introduction

Vasopressin (AVP, a neuropeptide) is a powerful regulator of various social behaviors across many species, from rodents to primates and humans (Thompson et al., 2006; Donaldson and Young, 2008; Insel, 2010). On the one hand, early animal studies have demonstrated that AVP is associated with defensive aggression (Ferris et al., 1986, 1997; Young et al., 1997; Semsar et al., 2001; Caldwell and Albers, 2004) and territorial behavior (Bielsky and Young, 2004; Meyer-Lindenberg et al., 2011; Caldwell, 2017). The dominant male voles have more AVP-immunoreactive neurons than subordinate one (Rosen et al., 2007; Qiao et al., 2014). In addition, the injection of AVP into anterior hypothalamus resulted in increased aggressive behaviors in male hamsters (Caldwell and Albers, 2004). On the other hand, AVP has been found to facilitate pair bonds (Winslow et al., 1993; Liu et al., 2001; Gobrogge et al., 2009) and paternal caregiving behaviors in males (for a review, see Goodson and Bass, 2001). Intranasal treatment of AVP induced affiliative responses toward females or a partner in male callicebus cupreus (Jarcho et al., 2011) and increased preference for the familiar partner in prairie voles (Cho et al., 1999). Furthermore, AVP is associated with increased vigilance to selective protection and affiliative behaviors (e.g., grooming) to the young (Carter, 1998; Kim et al., 2014).

Based on animal research, the past decade has witnessed an increasing interest in exploring the modulations of AVP on human social functioning. Similar to findings of animal studies, AVP could induce both aggression-related responses and social bonding among humans. Regarding the role of AVP in human aggression, it has demonstrated that the cereborspinal fluid level of AVP was positively correlated with the aggressive life histories in individuals with personality-disorder (Coccaro et al., 1998). Furthermore, intranasal AVP stimulated agonistic facial motor patterns and decreased friendliness ratings to same-sex facial expressions in men (Thompson et al., 2004, 2006). Lastly, intranasal AVP decreased the accuracy in inferring emotions from eyes of others (Uzefovsky et al., 2012). Regarding the role of AVP in social bonding, intranasal AVP promotes cooperation among strangers (Rilling et al., 2012, 2014; Feng et al., 2015; Brunnlieb et al., 2016), facilitates pair-bonding (Walum et al., 2008; Taylor et al., 2010), and supports fatherhood (Settersten, 2011; Kim et al., 2014).

Taken together, previous animal and human studies revealed seemingly contradictory roles of AVP in social behaviors, i.e., promoting aggression on one hand and facilitating social bonding on the other hand. Nevertheless, both aggression and social bonding are adaptive in particular environment. Therefore, it is possible that the role of AVP in social behaviors is context-dependent, closely related to specific targets (e.g., strangers, infants) that one is interacting with. The current work examined this hypothesis by investigating the potential effects of AVP in the processing of two different types stimuli, i.e., infant-related and adult-related cues. Specifically, in a randomized, placebo-controlled, double-blind event-related potential (ERP) study, male participants were presented with negative, neutral, and happy facial expressions as well as the specific contexts in which those expressions happened (e.g., “baby cried because of boredom” or “baby cried because of illness”). ERP signals were recoded for both contextual and facial cues. Participants were asked to indicate how much they were willing to approach to each target based on contextual and facial expression information.

Previous ERP studies on the processing of infant and adult cues have frequently revealed modulations of both early (e.g., N1, N170) and late temporal dynamics (e.g., P300, LPP) (Eimer and Holmes, 2007; Luo et al., 2010). Compared to neutral stimuli, negative infant cues induced larger N1 (Proverbio et al., 2006) and 170 amplitude in the early temporal stage (Proverbio et al., 2006; Rodrigo et al., 2011; Doi and Shinohara, 2012; Peltola et al., 2014; Ma et al., 2017; Rutherford et al., 2017) as well as more pronounced P300/LPP amplitude at the late temporal stage (Proverbio et al., 2006; Doi and Shinohara, 2012; Bernard et al., 2015; Malak et al., 2015; Ma et al., 2017). Likewise, positive infant stimuli compared to the neutral cues are associated shorter N1 latency (Peltola et al., 2014), larger N170 amplitudes (Bernard et al., 2015; Ma et al., 2017) at the early temporal stage, as well as larger P300 or later positive potential (LPP) at the late temporal stage (Proverbio et al., 2006; Doi and Shinohara, 2012; Bernard et al., 2015; Malak et al., 2015). However, contradictory findings have been also reported, such that the neutral infant cues were sometimes associated with shorter N170 latency (Ma et al., 2017) and larger prefrontal N2 amplitude (Proverbio et al., 2006) compared to emotional infant cues.

Regarding adult cues, relative to neutral stimuli, both negative (Eimer and Holmes, 2002; Balconi and Pozzoli, 2003; Batty and Taylor, 2003; Ashley et al., 2004; Holmes et al., 2005; Sun et al., 2017) and positive adult cues (Eimer and Holmes, 2007; Grasso et al., 2009; Calvo and Beltran, 2014; Morel et al., 2014; Neath-Tavares and Itier, 2016; Sun et al., 2017) are found to be associated with larger ERP responses at early temporal stage (e.g., <200 ms), including N1 (Eimer and Holmes, 2002; Eger et al., 2003; Bar-Haim et al., 2005; Luo et al., 2010) and N170 in occipital-temporal areas (Batty and Taylor, 2003; Caharel et al., 2005; Blau et al., 2007; Eimer and Holmes, 2007; Righart and De Gelder, 2008; Calvo and Beltran, 2014; Morel et al., 2014; Neath-Tavares and Itier, 2016; Sun et al., 2017), as well as enhanced late ERP responses, such as P300 and LPP (Eimer and Holmes, 2007; Grasso et al., 2009; Luo et al., 2010; Calvo and Beltran, 2014; Sun et al., 2017). However, other studies have identified that larger ERP amplitudes for neutral stimuli compared to the emotional adult cues, including P1 (Bar-Haim et al., 2005), N1 (Eimer and Holmes, 2002), N2 (Eimer and Holmes, 2002; Carretié et al., 2004), and LPP components (Krolak-Salmon et al., 2001). This finding could be attributed to the ambiguity or uncertainty of the neutral stimuli (Thompson et al., 2004, 2006). Indeed, neutral cues could be perceived as either threatening (Felmingham et al., 2003; Meyer et al., 2004; Cooney et al., 2006; Yoon and Zinbarg, 2008) or favorable stimuli (Krieglmeyer and Deutsch, 2013). Other study indicated that neutral faces was associated with a state of relaxation (Mignault and Chaudhuri, 2003), and therefore may weaken the vigilance from others.

Taken together, previous studies have identified modulations of both infant and adult cues at both early and late temporal stages. However, a majority of studies have mainly focused on the processing of infant and adult stimuli in a context-free manner, i.e., participants in these studies were exposed to the stimuli without contextual information. In our opinion, this is different from the way we perceive emotions of others in daily life, in which we often know the background information associated with others’ emotions. In addition, little is known about how emotions of infants and adults are modulated by AVP. The current work aimed to tackle these issues combining intranasal AVP treatment and an experimental design comprising both emotional expressions and contextual information.

In light of previous findings, we hypothesized that both infant and adult stimuli would modulate temporal dynamics at both early and late stages. These might be manifested as larger amplitude of early (e.g., N1, N170) and late (e.g., LPP) components induced by emotional stimuli compared to neutral faces. We further hypothesized that AVP would modulate the processing of infant and adult stimuli in a different manner, such as rendering neutral infant faces more approachable but neutral adult faces less approachable. Notably, however, it is difficult to derive specific hypotheses from the current literature about the effect of AVP at different temporal stages.

## Materials and Methods

### Participants

**FIGURE 1:**
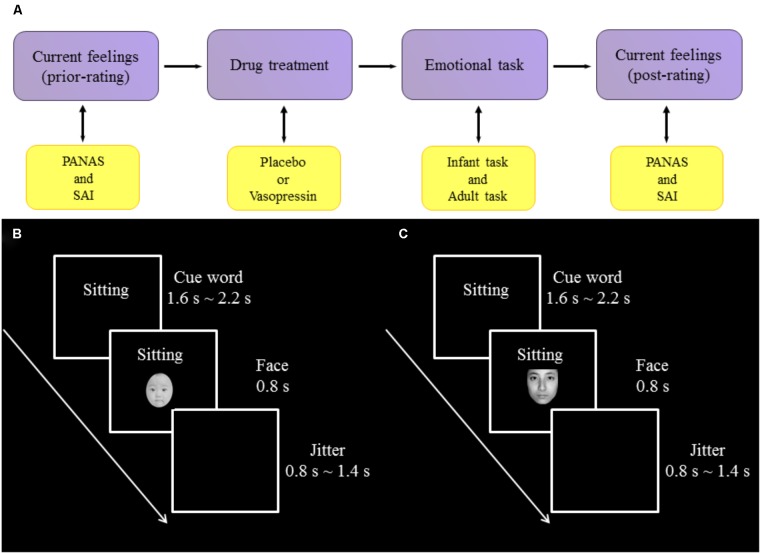
Experimental procedure and design. **(A)** The procedure of experiment. **(B)** One trial of infant task. **(C)** One trial of adult task.

Forty-eight males aged 18–26 years (mean age: 22.46, *SD* = 2.02) participated in the present study. They were randomly assigned to receive vasopressin (*n* = 24) or placebo (*n* = 24) treatment (**Figure 1A**). There was no significant difference between two groups with respect to age and education level among other demographic dimensions (**Table 1**). All participants had normal or corrected-to-normal vision, and did not have any history of psychiatric or neurological illness. Written informed consents were collected for all participants prior to the experiment. Participants were instructed to abstain from alcohol and caffeine on the day and from food and drink (except water) 2 h before drug administration. This study and the recruitment of participants were approved by Ethics Committee of Beijing Normal University and it was performed strictly in accordance with the approved guidelines. One participant from the placebo group was excluded from the ERP data analysis due to technical failure of electroencephalogram (EEG) recording.

**Table 1:** Demographics information from participants.

	Placebo group	AVP group		
				
	(*n* = 24)	(*n* = 24)		
				
	Mean (SD)	Mean (SD)	Two sample *t-test* (df = 46)	*p*-Value
Age	22.42 (1.95)	22.21 (2.00)	0.365	0.717
Height (cm)	174.97 (5.73)	174.50 (4.08)	0.325	0.746
Weight (kg)	71.27 (14.81)	67.88 (8.41)	0.977	0.334
Number of siblings	0.79 (1.44)	1.29 (1.30)	-1.260	0.214
Prior-Positive Affect Schedule	31.54 (6.16)	32.50 (5.90)	-0.551	0.585
Prior-Negative Affect Schedule	18.25 (6.42)	19.08 (8.77)	-0.376	0.709
Prior-State Anxiety	38.83 (7.81)	36.83 (11.35)	0.711	0.481
Post-Positive Affect Schedule	30.50 (7.52)	30.75 (6.60)	-0.122	0.903
Post-Negative Affect Schedule	20.00 (6.55)	20.63 (7.46)	-0.308	0.759
Post-State Anxiety	40.83 (9.38)	41.46 (12.18)	-0.199	0.843

	**Percentage (counts)**	**Percentage (counts)**	**Person Chi-square (df = 1)**	***p*-Value**

Handedness (L/R)	4.17% (1/23)	0% (0/24)	1.021	0.312
Relationship status (relationship/single)	41.17 (10/14)	33.33% (8/16)	0.356	0.551
Sexual orientation (non-heterosexual/heterosexual)	8.33% (2/22)	8.33% (2/22)	0.000	1.000
Only child (yes/no)	66.67% (16/8)	37.5% (9/15)	4.090	0.043
Experiences in taking care of child (yes/no)	58.33% (14/10)	70.83% (17/7)	0.820	0.365
Preferences in gender of one’s own baby (girl/boy)	54.17% (13/11)	41.67 (10/14)	0.751	0.386

	**Average rank (SD)**	**Average rank (SD)**	**Mann–Whitney test (Z)**	***p*-Value**

Education level	1.38 (0.49)	1.33 (0.56)	-0.490	0.624
Birth sequence	1.33 (1.05)	1.71 (0.81)	-2.592	0.010
Family income	2.54 (1.25)	2.13 (1.12)	-1.351	0.177
The loveliness of infants	3.71 (0.75)	3.5 (1.02)	-0.703	0.482


*The education level were rated for three ranks (1 = Bachelor, 2 = Master, 3 = Doctor). The family income included six ranks (1 = 1–6 millions/every year; 2 = 6–12 million/every year; 3 = 12–24 millions/years; 4 = 24–36 millions/years; 5 = 36–60 millions/years; 6 = 60–100 millions/years). The loveliness of infants were divided into five ranks (from 1 “*not at all*” to 5 “very much”).*

### Stimuli

Photographs of infant faces were adapted from online resources^1^. A total of 70 baby faces (35 boys and 35 girls) were selected, consisting of sad (*n* = 28), neutral (*n* = 14), and happy (*n* = 28) expressions. To validate the emotional category of infant faces, an additional group of 37 participants were recruited to rate the emotional valence (“*To what extent this baby faces made you pleased?*”), arousal (*“To what extent this baby faces made you excited?”*) on a nine-Likert scale, and to classify each face (*“Is this baby happy, sad, or neutral?”*). The results showed that three categories of infant faces (i.e., sad, neutral, and happy) showed significant differences in emotional valence [*F*(2,72) = 49.69, *p* < 0.001, $\eta_{p}^{2}$ = 0.58; happy = 6.20 ± 0.15; sad = 3.95 ± 1.71; neutral = 5.56 ± 0.095; pairwise comparisons, all *p* < 0.001], and arousal [*F*(2,72) = 28.29, *p* < 0.001, $\eta_{p}^{2}$ = 0.44; happy = 3.92 ± 1.89; sad = 5.48 ± 2.40; neutral = 3.94 ± 0.18; pairwise comparisons: sad/happy vs. neutral, *p* < 0.001, happy vs. sad, *p* > 0.05]. The results are consistent with the native Chinese Affective Face Picture System (Gong et al., 2011). In addition, the average classification accuracy reaches 97.10% for sad faces, 93.24% for happy faces, and 88.61% for neutral faces. Adult faces were selected from native Chinese Affective Face Picture System (Gong et al., 2011). Twenty-eight sad faces, 14 neutral faces, and 28 happy faces were selected, resulting in 70 adult faces (35 males and 35 females) in total. All images were normalized to the same luminance and contrast.

### Administration of AVP or Placebo

Intranasal administration has been widely applied in human and regarded as an effective method to bypass the blood–brain barrier and directly affect central nervous system (Waller et al., 2015; Rutherford et al., 2017). In present study, the AVP and placebo solutions were formulated by a trained research assistant who did not interact with subjects. The solutions were immediately sterilized before being transferred to sterile conical tube and stored at -80°C until use. On the day of the study, the drug was transferred to a nasal spray bottle. All solutions were administered intranasally. Both experimenters and participants were blind to the treatment participants received. The double-blind was also maintained by the research assistant. The AVP group self-administered 20 IU (Guastella et al., 2010, 2011; Rilling et al., 2012, 2017; Uzefovsky et al., 2012; Kenyon et al., 2013; Chen et al., 2016) arginine vasopressin (AVP) (ProSpec^2^). In each case, this required six nasal puffs to administer 0.5 ml of solution. The placebo group self-administered six nasal puffs of sterile saline. Participants were instructed to place the nasal applicator in one nostril and depress the lever until they felt a mist of spray in the nostril, to then breathe in deeply through the nose, and afterward to place the applicator in the other nostril and repeat the process. The experimental tasks began at about 80 min (79.77 ± 2.55) after drug administration (Born et al., 2002). After experiment, all participants were asked to report what (is it AVP or saline) they thought they received. The results showed the average accuracy reaches 45.83% for all participants, which did not significantly different from the random level [χ^2^(1) = 0.33, *p* = 0.56]. And there is no significant difference between placebo group and AVP group [45.83 vs. 45.83%, χ^2^(1) = 0.00, *p* = 1.00].

### Experimental Tasks

Experiments were conducted in a dimly lit and sound-attenuated chamber with a CRT monitor approximately 80 cm away from participants’ eyes. All participants performed an infant task and an adult task, the orders of which were counterbalanced across participants. In the infant task, participants were presented with sad (negative), neutral and happy (positive) expressions of infant photos as well as cue words indicating the situations in which those photos were putatively taken. Specifically, sad expressions were associated with “sick” (survival-related item) and “boring” (survival-unrelated item); neutral expressions with “sitting”; and happy expressions with “full” (survival-related item) and “playing” (survival-unrelated item). Each association was presented 14 times in each of three blocks of the task, resulting in 70 trials per block. On each trial, a cue word was presented (1600∼2200 ms) and followed by a facial expression (800 ms). Each trial was ended with a jitter (800∼1400 ms), during which a black screen was shown. Participants were instructed to respond to each facial expression by indicating how much they wanted to approach to the baby in the photo with a four-Likert scale (from 1 “*not at all*” to 4 “*very much*”).To reduce ocular artifact in the ERP analysis windows, participants were asked to blink only during the jitter. The procedure of infant task was illustrated in **Figure 1B**.

The adult task, also consisting of three blocks, was similar to the infant task except that (i) adult faces were presented and (ii) sad expressions were associated with “bereaved” (survival-related item) and “lost” (survival-unrelated item); neutral expressions with “sitting”; and happy expressions with “reunion” (survival-related item) and “traveling” (survival-unrelated item). The procedure of adult task was illustrated in **Figure 1C**.

### Post-rating

After experiment, the subjects were asked to rate their feelings of empathy ratings (“*To what extent you can feel the emotions of the baby or adult in this type of photos?*”), valence ratings (“*To what extent this type of photos made you pleased during experiment?*”), and arousal ratings (“*To what extent this type of photos made you excited during experiment?*”) in response to each emotional conditions (i.e., negative, neutral, and positive) with a nine-Likert scale (from 1 “*not at all*” to 9 “*very much*”).

### Mood Measurements

To evaluate any effects of AVP on mood or anxiety, participants also completed the Positive and Negative Affect Schedule (PANAS) (Watson et al., 1988) and State Anxiety (SAI) (Spielberger, 1983) before drug administration and at the end of experiment.

### Behavioral Data Analysis

The behavioral data analyses were conducted in the SPSS (IBM SPSS Statistics, v.21). *p*-Values were corrected for deviations according to Greenhouse–Geisser correction if necessary. Bonferroni correction was used for multiple comparisons unless noted otherwise. Notably, since we did not identify reliable differences in either behavioral or neural responses to survival-related and survival-unrelated conditions, these conditions were collapsed in both behavioral and ERP analyses. In addition, four participants were non-exclusively heterosexuality. Therefore, we added supplementary analyses by excluding these four subjects. These analyses revealed similar findings and were illustrated in **Supplementary Table S1**.

#### Main Tasks

For the emotional task, participants’ approaching ratings to each person in the photo and their response times (RTs) were analyzed. Four-way repeated measures analysis of variances (ANOVAs) were implemented with Drug (placebo vs. AVP) and Tasks (infant task vs. adult task) as the between-subjects factors, Emotional valence (negative vs. neutral vs. positive) and Gender (boy/male vs. girl/female) as within-subjects factors.

#### Post-ratings

Three-way repeated measures ANOVAs were implemented with Drug (placebo vs. AVP), Tasks (infant task vs. adult task) and Emotional valence (negative vs. neutral vs. positive) on the average scores of empathy, valence, and arousal ratings.

#### Mood Measurements

Two samples *t*-test were implemented to examine effects of drug treatments (placebo vs. AVP) on state feelings measured with PANAS and State Anxiety before and at the end of the experiment.

### EEG Recording

The EEG was recorded from 64 scalp sites using electrodes mounted in an elastic cap (Compumedics, Houston, TX, United States), with an online reference to the left mastoid. The horizontal electroencephalogram (HEOG) was recorded with two electrodes placing laterally to the right and left eyes. The vertical electroencephalogram (VEOG) was recorded with electrodes placed above and below the left eye. All inter-electrode impedances were maintained below 10 kΩ. The EEG and EOG were amplified using a 0.05–100 Hz band-pass and continuously sampled at 500 Hz in each channel for off-line analysis. EEGs were first re-referenced to the algebraic average of left mastoid and right mastoid and then to the average of all of the electrodes. Data were then corrected for ocular artifacts with algorithm implemented in the Neuroscan Edit 4.5 software (Compumedics, Houston, TX, United States). The resulting data were then epoched from -200 to 800 ms relative to the onset of stimuli (i.e., cue words or faces), and baseline corrected from -200 to 0 ms. Afterward, EEG data were low-pass filtered below 30 Hz. Artifact rejection was performed for all of the EEG channels, and the rejection criteria was ±80 μV.

### Data Reduction and Analysis

Event-related potentials elicited by both cue words and faces were analyzed. Based on the inspection of grand-averaged ERP waveforms and previous studies of emotion processing (Luo et al., 2010; Gong et al., 2011), three different ERP components, anterior N1, N170 (posterior N1) and LPP were measured in the current study. Different sets of electrodes were chosen for the area measurements of these components. Specifically, fronto-central electrodes (FC3/FC4/FC5/FC6) (Penga et al., 2012) were chosen for anterior N1 component (120–160 ms); occipito-temporal electrodes (P7/P8/PO7/PO8/O1/O2) (Luo et al., 2010; Penga et al., 2012) were chosen for N170 component (180–220 ms); centro-parietal electrodes (C5/C6/CP5/CP6/P5/P6) (Gootjes et al., 2011) were chosen for LPP component (300–700 ms). The mean of amplitudes of these components were then analyzed in repeated measures ANOVAs with the factors of Drug (placebo vs. AVP) and Tasks (infant task vs. adult task) as the between-subjects factors, Emotional valence (negative vs. neutral vs. positive), Hemisphere (left vs. right) as well as Electrode as within-subjects factors. The factor of Gender (boy/male vs. girl/female) was only considered for the analyses of face-elicited ERPs, since no gender information could be obtained during the presentation of cue words. The EEG data analyses were also conducted in the SPSS (IBM SPSS Statistics, v.21). *p*-Values were corrected for deviations according to Greenhouse–Geisser correction if necessary. Bonferroni correction was used for multiple comparisons unless noted otherwise. Partial eta-squared $\eta_{p}^{2}$ values were provided to demonstrate effect size where appropriate, such that 0.05 represents a small effect, 0.10 represents a medium effect, and 0.20 represents a large effect (Cohen, 1973).

## Results

### Mood Measurements

For state mood and anxiety measured before drug treatment or after experiment, there was no significant difference between placebo and AVP groups (**Table 1**).

### Emotional Task

#### Behavior Results

**FIGURE 2:**
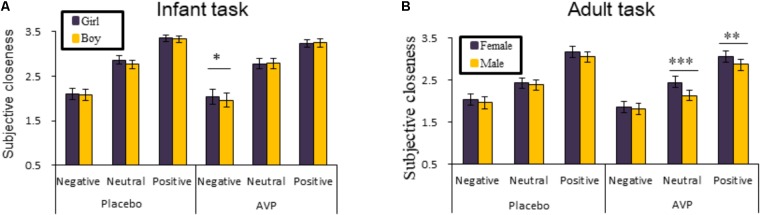
Behavioral results of the emotional task. The mean ratings of approaching ratings as a function of Drug treatment, Gender and Emotional valence in infant task and adult task **(A,B)**. Four-way repeated measures ANOVAs on the RTs did not reveal any significant effects related to Drug treatment. ^∗^*p* < 0.05; ^∗∗^*p* < 0.01; ^∗∗∗^*p* < 0.001.

**FIGURE 3:**
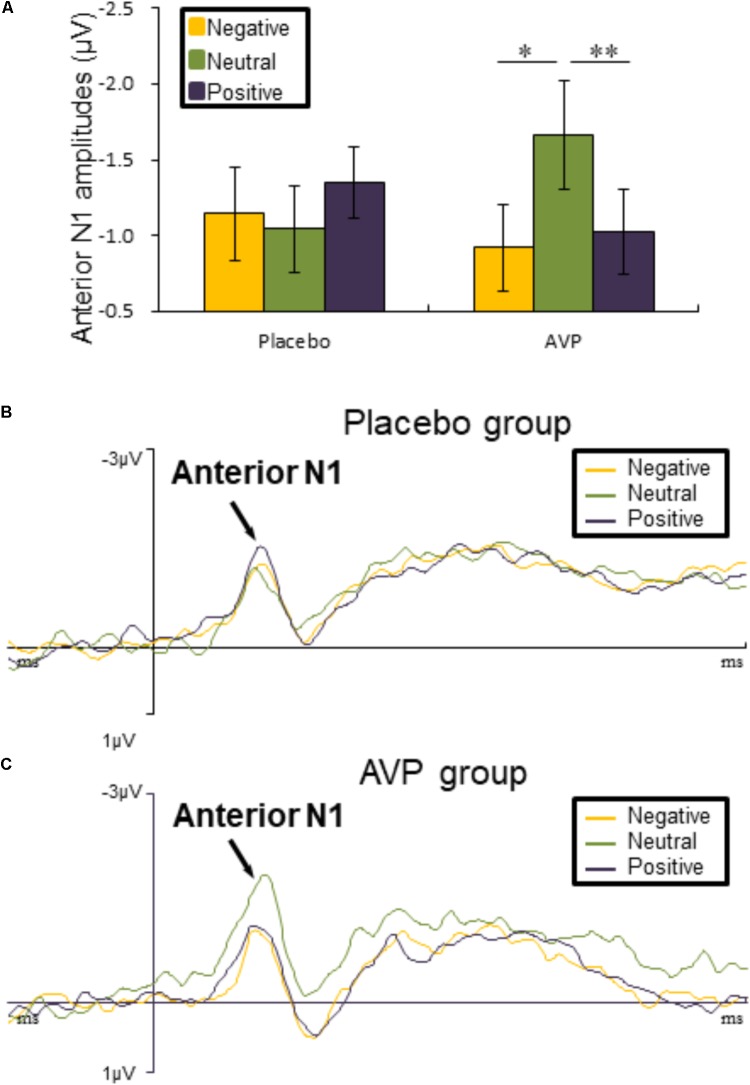
Event-related potential (ERP) results related to cue words. **(A)** Bar graph illustrated the mean anterior N1 amplitude as a function of Drug treatment and Emotional valence in both tasks. **(B,C)** The grand average ERPs over left hemisphere (FC3 and FC5) evoked by negative, neutral, and positive words in the placebo and AVP group. ^∗^*p* < 0.05; ^∗∗^*p* < 0.01; ^∗∗∗^*p* < 0.001.

The behavioral results of emotional tasks were shown in **Figure 2**. Four-way repeated measures ANOVAs on the approaching ratings revealed a significant interaction of Drug × Tasks × Emotional valence × Gender [*F*(2,92) = 8.85, *p* = 0.0003, $\eta_{p}^{2}$ = 0.161]. The *post hoc* comparisons reveal the statistical difference between Gender, such that AVP increased subjective approaching ratings to girl faces compared boy faces exhibiting negative expressions (*p* = 0.048) in infant task (**Figure 2**). In adult task, such that AVP treatment decreased subjective approaching ratings to neutral (*p* = 0.000) and positive (*p* = 0.004) males faces compared to Emotional valence-matched female faces. In the placebo group, no significant differences were identified (**Figure 3**). The *post hoc* comparisons revealed no statistical difference between Drug (AVP vs. placebo) in all conditions (*p* > 0.05) and no statistical effects between Tasks (infant task vs. adult task) related to Drug treatment (*p* > 0.05). In addition, the Four-way repeated measures ANOVAs on the RTs did not reveal any significant effects related to drug treatment. There were no significant effects related to drug treatment on empathy, valence, and arousal ratings measured after the experiment (**Table 2**).

**Table 2:** Subjective ratings of empathy, valence, and arousal after experiment as a function of Drug treatment, Task, and Emotional valence.

			Placebo group	AVP group
				
			(*n* = 24)	(*n* = 24)
				
			Mean (SD)	Mean (SD)
Infant task	Empathy ratings	Negative	6.08 (2.04)	6.00 (2.05)
		Neutral	5.46 (1.79)	4.88 (1.94)
		Positive	6.85 (1.27)	5.79 (1.92)
	Valence ratings	Negative	2.79 (0.94)	3.06 (1.43)
		Neutral	5.63 (0.97)	5.33 (1.40)
		Positive	7.40 (1.26)	6.65 (1.40)
	Arousal ratings	Negative	5.88 (2.08)	5.77 (1.91)
		Neutral	3.92 (1.44)	4.25 (1.51)
		Positive	3.52 (1.15)	4.00 (1.56)
Adult task	Empathy ratings	Negative	5.73 (1.99)	6.25 (1.94)
		Neutral	4.79 (1.77)	5.04 (1.92)
		Positive	6.88 (1.25)	7.04 (1.28)
	Valence ratings	Negative	2.77 (0.93)	2.46 (1.48)
		Neutral	4.88 (1.23)	5.38 (1.66)
		Positive	6.65 (1.44)	6.92 (1.43)
	Arousal ratings	Negative	5.81 (1.75)	6.52 (1.70)
		Neutral	3.67 (1.79)	4.17 (1.43)
		Positive	3.92 (1.44)	4.19 (1.44)


Two-way repeated measures ANOVAs were implemented with Drug (placebo vs. AVP) and Emotional valence (negative vs. neutral vs. positive) on the average scores of empathy, valence and arousal ratings, which did not shown any significant effect related to drug treatment.

### ERP Components Evoked by Cue Words

#### Anterior N1

The five-way repeated measures ANOVAs on anterior N1 amplitude revealed a significant interaction of Drug × Emotional valence × Hemisphere [*F*(2,90) = 4.50, *p* = 0.014, $\eta_{p}^{2}$ = 0.091], such that anterior N1 amplitude over left hemisphere to both negative (*p* = 0.012) and positive (*p* = 0.006) words were attenuated compared to neutral conditions in the AVP group (**Figures 3A,C**). In the placebo group, however, no significant differences were identified (**Figures 3A,B**). The *post hoc* comparisons revealed no statistical difference between Drug (AVP vs. placebo) in all conditions (*p* > 0.05) and no statistical effects between Tasks (infant task vs. adult task) related to Drug treatment (*p* > 0.05).

#### N170

The five-way repeated measures ANOVAs on N170 amplitude revealed neither main effect not interactions related to drug treatment.

#### Later Positive Potential

**FIGURE 4:**
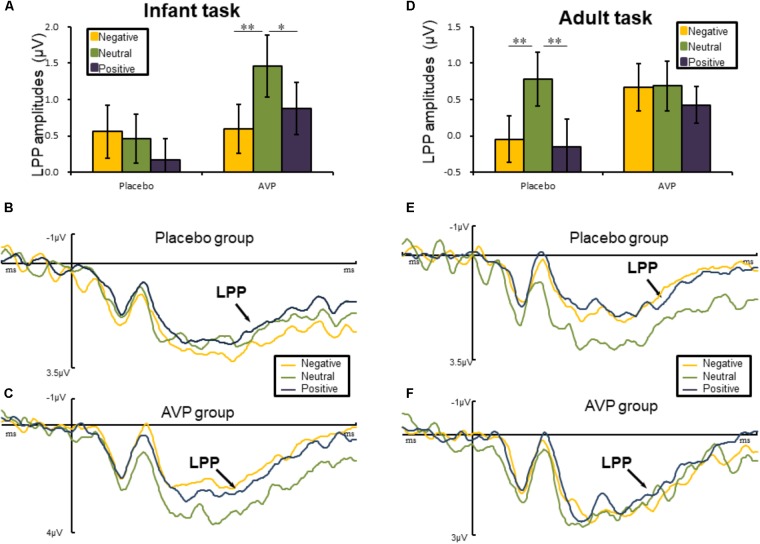
Event-related potential results related to cue words. **(A,D)** Bar graph illustrated the mean later positive potential (LPP) amplitude as a function of Drug treatment and Emotional valence in infant and adult tasks. **(B,C)** In infant task, the grand average ERPs over right hemisphere (C6, CP6, and P6) evoked by negative, neutral, and positive words in the placebo and AVP group. **(E,F)** In adult task, the grand average ERPs over right hemisphere (C6, CP6, and P6) evoked by negative, neutral, and positive words in the placebo and AVP group. ^∗^*p* < 0.05; ^∗∗^*p* < 0.01; ^∗∗∗^*p* < 0.001.

The five-way repeated measures ANOVAs on LPP amplitude revealed a significant interactions of Drug × Tasks × Emotional valance × Hemisphere [*F*(2,180) = 3.45, *p* = 0.036, $\eta_{p}^{2}$ = 0.071]. *Post hoc* comparisons reveals that in infant task, the LPP amplitude over right hemisphere to both negative (*p* = 0.01) and positive (*p* = 0.028) words were reduced compared to the neutral words in AVP group (**Figures 4A,C**). In the placebo group, however, no significant differences were identified (**Figures 4A,B**). In adult task, *post hoc* comparisons revealed that LPP amplitude of both negative (*p* = 0.006) and positive (*p* = 0.002) over right hemisphere were attenuated compared to neutral words (**Figures 4D,E**) in placebo group. However, in the AVP group, no significant differences were identified (**Figures 4D,F**). The *post hoc* comparisons revealed no statistical difference between Drug (AVP vs. placebo) in all conditions (*p* > 0.05) and no statistical effects between Tasks (infant task vs. adult task) related to Drug treatment (*p* > 0.05).

### ERP Components Evoked by Faces

#### Anterior N1

The six-way repeated measures ANOVAs on LPP amplitude revealed neither main effect not interactions related to drug treatment.

#### N170

**FIGURE 5:**
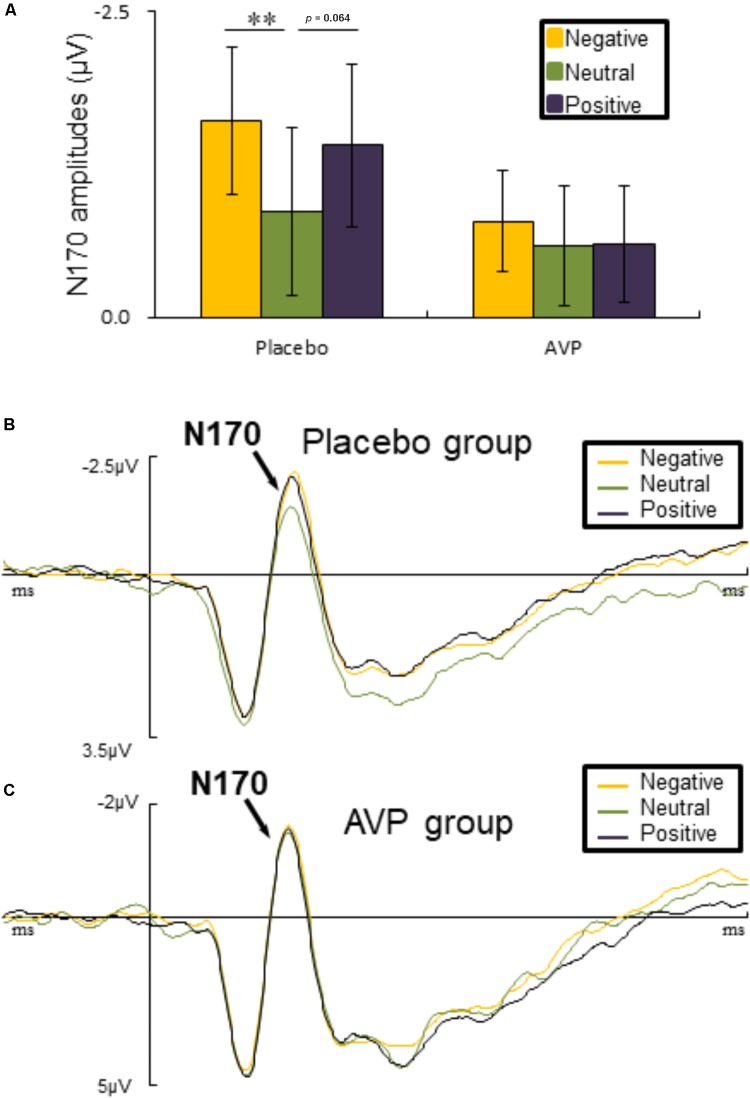
Event-related potential results related faces. **(A)** Bar graph illustrated the mean N170 amplitude as a function of Drug treatment and Emotional valence in both tasks. **(B,C)** The grand average ERPs over left hemisphere (O1, P7, and PO7) evoked by negative, neutral, and positive faces in placebo and AVP group. ^∗^*p* < 0.05; ^∗∗^*p* < 0.01; ^∗∗∗^*p* < 0.001.

The six-way repeated measures ANOVAs on N170 amplitude revealed a significant interaction of Drug × Emotional valence × Hemisphere [*F*(2,90) = 5.023, *p* = 0.009, $\eta_{p}^{2}$ = 0.1], such that negative faces (*p* = 0.004) elicited lager N170 amplitude over the right hemisphere compared to the neutral faces in placebo group. There was also a marginal difference showing that positive faces evoked larger N170 amplitudes over the left hemisphere than neutral faces (*p* = 0.064) (**Figures 5A,B**). However, in the AVP group, no significant differences were founded (**Figures 5A,C**). The *post hoc* comparisons revealed no statistical difference between Drug (AVP vs. placebo) in all conditions (*p* > 0.05) and no statistical effects between Tasks (infant task vs. adult task) related to Drug treatment (*p* > 0.05).

#### Later Positive Potential

The six-way repeated measures ANOVAs on LPP amplitude revealed neither main effect not interactions related to drug treatment.

## Discussion

The current study investigated how AVP modulated the processing of infant and adult cues and the time course of these modulations. During EEG recording, participants were instructed to rate subjective approaching ratings to infant and adult faces in specific contexts informed by cue words. Behaviorally, AVP treatment increased approaching ratings to neutral and positive other-gender adult faces compared to emotional-matched same-gender adult faces, and to negative girl faces compared to negative boy faces. At the neural level, effects related to AVP were present at early temporal stages, as respectively reflected by anterior N1 (120–160 ms), Occipital N170 (190–230 ms) and extended to late temporal stages as reflected by LPP (300–700 ms). In the followings, we would discuss the roles of AVP at behavior (e.g., subjective approaching ratings) and neural levels.

### Behavioral Results

Among male participants in the current study, intranasal AVP decreased approaching ratings to same-gender adult faces and increased approaching ratings to other-gender adult face. Importantly, those modulations were limited to the neutral and positive faces. Therefore, the current findings are in line with the hypothesis that the modulations of AVP are context-dependent. In accordance, previous studies have reported that AVP facilitated both recognition of sexual cues (Walum et al., 2008; Taylor et al., 2010; Guastella et al., 2011; Rilling et al., 2017) and negative social interaction to same-gender faces in human males (Thompson et al., 2004, 2006). For example, men perceived female faces as more attractive than male faces after intranasal AVP treatment (Price et al., 2017). In contrast, intranasal AVP decreased approaching ratings to happy same-gender faces in men (Thompson et al., 2006). Taken together, it is possible that both aggression and affiliation could be simultaneously elicited by AVP for a certain social context (Caldwell and Albers, 2015).

### ERP Components Evoked by Cue Words

For both infant and adult cues, AVP treatment increased anterior N1 response to neutral compared to emotional cues at the early stage, an effect that was absent in the placebo group. The anterior N1 has been described as rapid but crude processing of face stimuli (Grasso et al., 2009) and faster responses to probes (Luck et al., 1994). Therefore, enhanced N1 response to neutral adult and infant cues might reflect more attentional resources allocated to those stimuli. Distinct motivations might be underlie preferential processing of neutral adults and infant cues during the early temporal stages. In particular, neutral adult cues could be perceived as potentially threatening stimuli due to uncertain and ambiguity properties of those stimuli (Felmingham et al., 2003; Meyer et al., 2004; Thompson et al., 2004, 2006; Cooney et al., 2006; Yoon and Zinbarg, 2008). Therefore, preferential processing of neutral adult cues might reflect rapid detection of potential threats in the environment. In contrast, neutral infant cues often drive approaching intentions from others (Proverbio et al., 2011) and have been associated with reword-related processing (Strathearn et al., 2008). In this regard, preferential processing of these stimuli might be motivated by protection-related intentions. In summary, it is conceivable that AVP facilitate rapid allocation of attention to adult and infant cues due to defensive vigilance and intentions for caregiving, respectively.

This conjecture is consistent with the differential effects of AVP at the late temporal stages. That is, AVP treatment resulted in larger LPP amplitude to infant cues but not adult cues. Considering the critical role of the LPP in maintaining sustained attention (Cuthbert et al., 2000; Hajcak and Olvet, 2008), our findings suggest that AVP facilitates prolonged attention to infant cues but not adult cues. These findings could be attributed to the reason that caregiving arguably requires sustaining attention rather than transit attention to detect more detailed needs for infants. In contrast, humans are well-equipped in detecting and processing potential threats in a fast and automatic manner (Öhman, 2002, 2005; Santesso et al., 2008). Indeed, human responses to threats are fast, often occurring within 100 ms post-stimulus onset (Liu et al., 2002; Sun et al., 2012), so that threatening situations can be detected and avoided quickly (Olofsson et al., 2008).

### ERP Components Evoked by Faces

For both infant and adult faces, emotional faces evoked larger N170 amplitudes than neutral ones in placebo group, in line with substantial previous studies (Proverbio et al., 2006; Rodrigo et al., 2011; Doi and Shinohara, 2012; Peltola et al., 2014; Bernard et al., 2015; Ma et al., 2017; Rutherford et al., 2017). These findings has been interpreted as the mandatory allocation of attentional resources to biologically relevant stimuli (Fehm-Wolfsdorf et al., 1984; Pietrowsky et al., 1996; Batty and Taylor, 2003; Carretié et al., 2004; Rossignol et al., 2005; Luo et al., 2010; Morel et al., 2014; Patel et al., 2015; Sun et al., 2017). However, the differential processing of emotional and neutral faces was eliminated by the treatment of AVP. This is in line with the idea that AVP may bolster the processing of ambiguous events (e.g., neutral faces).

There are several limitations in present study. Firstly, we adopted a between-subjects design, which might retain the individual variations and therefore might attenuate the statistical power. Notably, however, the AVP and placebo groups are reasonably matched in terms of the demographic information, mood measurements, and post-ratings. Secondly, there are two participants reporting themselves as non-exclusively heterosexual in each group. Although whether AVP effects are dependent on sexual orientation is an important question, the current study cannot systematically address this question. This intriguing topic awaits to be explored in future studies. Finally, the small sample sizes should be noted and await replication by greater sample in further study.

In summary, based on previous observations on the AVP in both aggression and social bonding, here we examine context-dependent effects of AVP on the processing of adult and infant cues among males. The results of present study indicate that the modulation of AVP were context-based. At early temporal state, intranasal AVP induced rapid attention to both adult and infant cues, whereas AVP treatment only facilitated sustained attention to infant cues but not adult cues. Different effects of AVP on the neural dynamics underlying the processing of adult and infant cues might be attributed to different neuropsychological mechanisms, i.e., rapid detection of threat in response to adult cues whereas sustained attention for potential caregiving in response to infant cues.

## Author Contributions

XW, PX, CF, and Y-JL: conception and drafting of the work.

## Conflict of Interest Statement

The authors declare that the research was conducted in the absence of any commercial or financial relationships that could be construed as a potential conflict of interest.

## Footnotes

1 https://www.vcg.com/

2 https://www.prospecbio.com/Vasopressin
