# Supplementary material for: Differential Effects of Intranasal Vasopressin on the Processing of Adult and Infant Cues: An ERP Study
Source: Front Hum Neurosci. 2018 Aug 15;12:329. doi: 10.3389/fnhum.2018.00329 (PMC6104155; doi:10.3389/fnhum.2018.00329)
Supplement: Supplementary file 1 [file Table_1.DOCX]

**Table S1. The behavioral and ERP results of exclusive-heterosexual subjects.**

| **Data analysis** | | **Results** |
| --- | --- | --- |
| Approaching ratings | Four-way repeated measures ANOVAs were implemented with Drug×Tasks×Emotional valence×Gender on approaching ratings | Four-way interaction of Drug×Tasks×Emotional valence×Gender (*F* (2, 84) =7.73, *p*=0.001, *η^2^_p_*=0.155). Post-hoc comparisons: firstly, the approaching ratings for negative girl faces were significantly higher than that for negative boy faces (*p*=0.04) in AVP group; secondly, the approaching ratings for neutral (*p*=0.000) and positive (*p*=0.024) women faces were significantly higher than that for emotional-matched men faces in AVP group, the effects were absent in placebo group. The post-hoc comparisons revealed no statistical difference between Drug (AVP *vs.* Placebo) in all conditions (*p*>0.05) and no statistical effects between Tasks (infant task *vs.* adult task) related to Drug treatment (*p*>0.05). |
| N1 evoked by cue words | Five-way repeated measures ANOVAs were implemented with Drug×Tasks×Emotional valence×Hemisphere×electrodes on N1 amplitude evoked by cue words | Three-way interaction of Drug×Emotional valence×Hemispthere (*F* (2, 82) =3.15, *p*=0.048, *η^2^_p_*=0.071). Post-hoc comparisons: the N1 amplitude over left hemisphere evoked by both negative (*p*=0.029) and positive (*p*=0.036) words were smaller than by neutral words in AVP group, the effect was absent in placebo group. The post-hoc comparisons revealed no statistical difference between Drug (AVP *vs.* Placebo) in all conditions (*p*>0.05) and no statistical effects between Tasks (infant task *vs.* adult task) related to Drug treatment (*p*>0.05). |
| N170 evoked by cue words | Five-way repeated measures ANOVAs were implemented with Drug×Tasks×Emotional valence×Hemisphere×electrodes on N170 amplitude evoked by cue words | The five-way repeated measures ANOVAs on N170 amplitude revealed neither main effect not interactions related to drug treatment. |
| LPP evoked by cue words | Five-way repeated measures ANOVAs were implemented with Drug×Tasks×Emotional valence×Hemisphere×electrodes on LPP amplitude evoked by cue words | Four-way interaction of Drug×Task×Emotional valence×electrodes (*F* (4,164) =3.726, *p*=0.006, *η^2^_p_*=0.083). Post-hoc comparisons: firstly, the LPP amplitude of P5 and P6 evoked by neutral words of infant task were significantly larger than by neutral words of adult task in AVP group (*p*=0.01), the effect was absent in placebo group. Secondly, the LPP amplitude evoked by negative words of infant task were significantly larger than emotion-matched words of adult task in placebo group (*p*=0.034), the effect was absent in AVP group. Finally, in infant task but not adult task, AVP increased the LPP amplitude of C5 and C6 electrodes evoked by negative words compared to placebo group (*p*=0.03). |
| N1 evoked by faces | Six-way repeated measures ANOVAs were implemented with Drug×Tasks×Emotional valence×Gender×Hemisphere×electrodes on N1 amplitude evoked by faces | The six-way repeated measures ANOVAs on N1 amplitude revealed neither main effect not interactions related to drug treatment. |
| N170 evoked by faces | Six-way repeated measures ANOVAs were implemented with Drug×Tasks×Emotional valence×Gender×Hemisphere×electrodes on N170 amplitude evoked by faces | Three-way interaction of Drug×Emotional valence×Hemispthere (*F* (2, 164) =4.95, *p*=0.009, *η^2^_p_*=0.11). Post-hoc comparisons: the negative (*p*=0.004) and positive (*p*=0.032) faces elicited larger N170 amplitudes over left hemisphere than neutral faces in placebo group regardless of infant or adult task, the effects were absent in AVP group. The post-hoc comparisons revealed no statistical difference between Drug (AVP *vs.* Placebo) in all conditions (*p*>0.05) and no statistical effects between Tasks (infant task *vs.* adult task) related to Drug treatment (*p*>0.05). |
| LPP evoked by faces | Six-way repeated measures ANOVAs were implemented with Drug×Tasks×Emotional valence×Gender×Hemisphere×electrodes on LPP amplitude evoked by faces | The six-way repeated measures ANOVAs on LPP amplitude revealed neither main effect not interactions related to drug treatment. |
